# An efficient and reproducible *Agrobacterium*-mediated transformation method for hexaploid wheat (*Triticum aestivum* L.)

**DOI:** 10.1186/s13007-019-0503-z

**Published:** 2019-10-26

**Authors:** Sadiye Hayta, Mark A. Smedley, Selcen U. Demir, Robert Blundell, Alison Hinchliffe, Nicola Atkinson, Wendy A. Harwood

**Affiliations:** Department of Crop Genetics, John Innes Centre, Norwich Research Park, Norwich, Norfolk NR4 7UH UK

**Keywords:** Wheat, *Triticum aestivum*, Genetic modification, *Agrobacterium tumefaciens*, Immature embryo

## Abstract

**Background:**

Despite wheat being a worldwide staple, it is still considered the most difficult to transform out of the main cereal crops. Therefore, for the wheat research community, a freely available and effective wheat transformation system is still greatly needed.

**Results:**

We have developed and optimised a reproducible *Agrobacterium*-mediated transformation system for the spring wheat cv ‘Fielder’ that yields transformation efficiencies of up to 25%. We report on some of the important factors that influence transformation efficiencies. In particular, these include donor plant health, stage of the donor material, pre-treatment by centrifugation, vector type and selection cassette. Transgene copy number data for independent plants regenerated from the same original immature embryo suggests that multiple transgenic events arise from single immature embryos, therefore, actual efficiencies might be even higher than those reported.

**Conclusion:**

We reported here a high-throughput, highly efficient and repeatable transformation system for wheat and this system has been used successfully to introduce genes of interest, for RNAi, over-expression and for CRISPR–Cas9 based genome editing.

## Background

Hexaploid wheat (*Triticum aestivum* L.) is one of the “big three” cereal crops after maize (*Zea mays*) and rice (*Oryza sativa*) [1–3]. It is unrivalled in its geographic range of cultivation and accounts for approximately 20% calorific value and 25% of daily protein intake of the world’s population [1]. Wheat has arguably, more influence on global food security than any other crop [4]. In spite of wheats global importance, it has lagged behind other main cereals, primarily rice and maize, in the development of genomic tools for its improvement and is still considered the most challenging of the major cereals to transform [5, 6].

In the early 1990’s the first report was published of transgenic wheat being produced by direct DNA transfer using particle bombardment (biolistics) of embryogenic callus tissue [7]. The first reported *Agrobacterium*-mediated transformation of wheat was to follow in 1997 [8]. Nevertheless, in spite of this first promising report, biolistic-mediated transformation of wheat remained the method of choice for some considerable time, as wheat transformation via *Agrobacterium* continued to be challenging and inefficient [9]. Reports in the literature of *Agrobacterium*-mediated wheat transformation generally describe low transformation efficiencies of around 5%. Efficient wheat transformation via an *in planta Agrobacterium*-mediated inoculation method was reported by Risacher et al. [10] however, this methodology required specialist skills and has not been widely adopted. Another efficient patented transformation system is available through licence from Japan Tobacco Inc (http://www.jti.co.jp), licenced as two systems, the basic PureIntro™ and the more advanced PureUpgrade™. Ishida et al. [11], described the process, and reported efficiencies of 40–90% however, our lab and others (personnel communication) have not been able to replicate the process based on the published information. It appears that the specialist training provided to laboratories licensing the technology is a prerequisite to successful reproduction of the method and/or specialist vectors are required. Therefore, a robust, reproducible and transferrable wheat transformation system that is widely available to the research community is still needed.

For any transformation method the procedure can be easily split into two component phases: those that enable efficient T-DNA transfer and incorporation into the plant genome and those that allow the selection of transformed cells and regeneration of whole transgenic plants [12]. In wheat, some factors affecting T-DNA transfer include; the binary vector used in conjunction with *Agrobacterium* strain, the inclusion of additional *vir* genes, pre-treatments of embryos, *Agrobacterium* inoculation and co-cultivation. The key factors influencing wheat regeneration in vitro include; the cultivar used, quality and health of donor material, stage of immature embryos, handling of material, and media composition including all components from nutrients to gelling agents and phytohormones [9, 13, 14].

In this present study we report a reproducible *Agrobacterium*-mediated transformation system for the spring wheat Fielder that yields transformation efficiencies up to 25%. This system has been widely used to introduce genes of interest as well as for CRISPR/Cas9 based genome editing [15].

## Materials and methods

### Plant material

Seeds of the spring wheat (*Triticum aestivum* L.) cv ‘Fielder’ were sown at weekly intervals in a mixture of peat and sand (85% fine grade peat, 15% washed grit, 4 kg m^−3^ maglime, 2.7 kg m^−3^ Osmocote (3–4 months), 1 kg m^−3^ PG Mix 14-16-18 + Te 0.02% and wetting agent). They were initially sown in 5 cm diameter pots and after approximately 4 weeks the germinated plants were transferred into 13 cm diameter pots containing John Innes Cereal Mixture (40% medium grade peat, 40% sterilised loam (soil), 20% washed horticultural grit, 3 kg m^−3^ maglime, 1.3 kg m^−3^ PG mix 14-16-18 + Te base fertiliser, 1 kg m^−3^ Osmocote mini 16-8-11 2 mg + Te 0.02%, and wetting agent) for continued development. Plants were grown in controlled growth chambers (Conviron Europe Ltd) at 20 ± 1 °C day and 15 ± 1 °C night temperatures, 70% humidity with light levels of 800 μmol m^−2^ s^−1^ provided by fluorescent tubes and tungsten lighting. At any stage of growth, the donor plants were not sprayed with insecticides or fungicides. In addition, particular care was taken to restrict unnecessary staff access to the controlled environment rooms, hairnets and designated lab coats were maintained in a freezer (− 20 °C) to reduce the risk of spreading pathogens.

### Immature embryos isolation

**Fig. 1:**
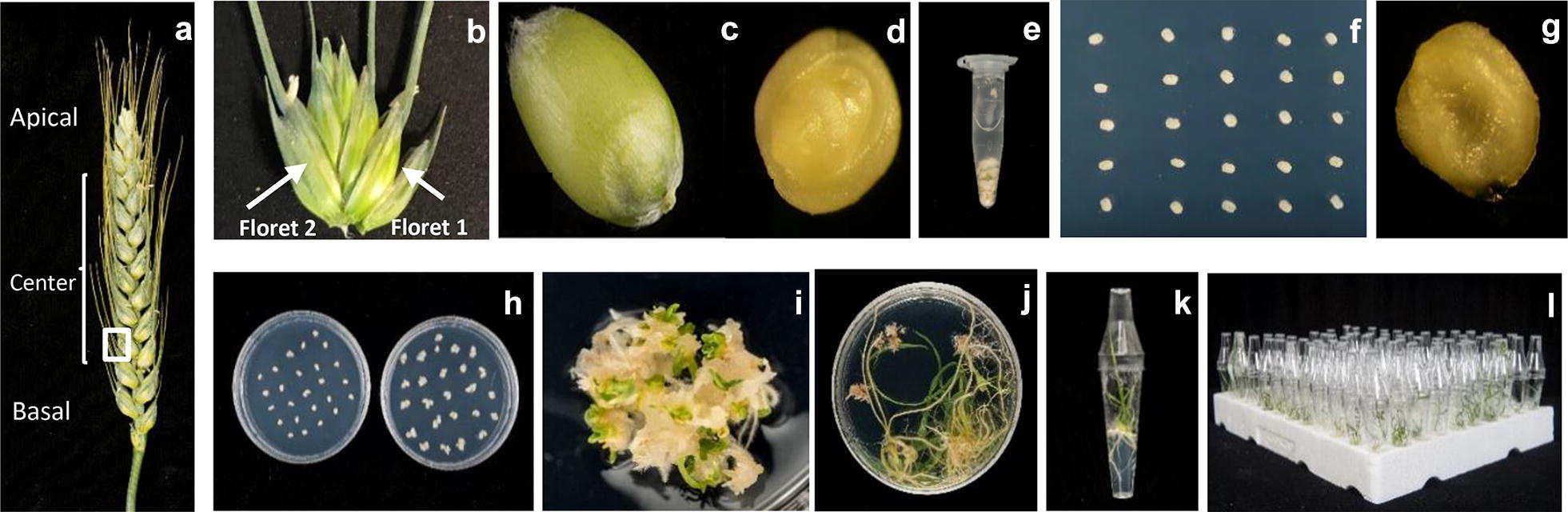
**a**–**c** Selection of wheat spikes and immature embryos at the correct stage, **d** isolated immature embryo, **e** immature embryos in Eppendorf tube containing 1 mL WIM, **f** immature embryos on co-cultivation medium, **g** immature embryo with the embryonic axis removed before transferring to resting medium, **h** callus induction on Selection 1 and Selection 2 media, **i** transformed callus starting to green and produce small shoots, **j** regenerated shoots with visibly strong roots, **k** transgenic wheat plant transferred to culture tube showing strong root system in hygromycin containing medium, **l** transgenic wheat plants before transferring to soil

Wheat spikes were collected approximately 14 days post anthesis (dpa), when the immature embryos (IE) were 1–1.5 mm in diameter (Fig. 1c, d) and early milk stage GS73 [16]. Kernels from floret 1 and 2 on central spikelet (Fig. 1a, b) were used for transformation. The awns were cut off the ears approximately 3–5 mm from the grain. The seed coat can be removed but this was not essential unless contamination problems are encountered. The immature grains were separated from the ear and placed in a 150 mL Sterilin jar. Within a laminar airflow cabinet under aseptic conditions, the grains were surface sterilised using 70% ethanol (v/v) for 1 min, given 1 rinse with sterile distilled water, followed by 7 min in 10% (v/v) sodium hypochlorite (Fluka 71696). The grains were then washed 3 times with sterile distilled water.

All subsequent operations were performed under sterile conditions in a laminar flow hood. Embryos were isolated from the immature grains using fine forceps under a dissecting microscope. Approximately 100 embryos were put into two 1.7 mL Eppendorf tubes (Fig. 1e) containing 1 mL wheat inoculation medium (WIM) modified from [11] containing 0.44 mg L^−1^ Murashige and Skoog (MS) [17] plant salt base (Duchefa M0222), 10 g L^−1^ glucose, 0.5 g L^−1^ 2-(*N*-morpholino) ethanesulfonic acid (MES), 0.05% Silwet L-77 and 100 µM Acetosyringone (AS) added fresh just before use.

### Construct assembly

**Fig. 2:**
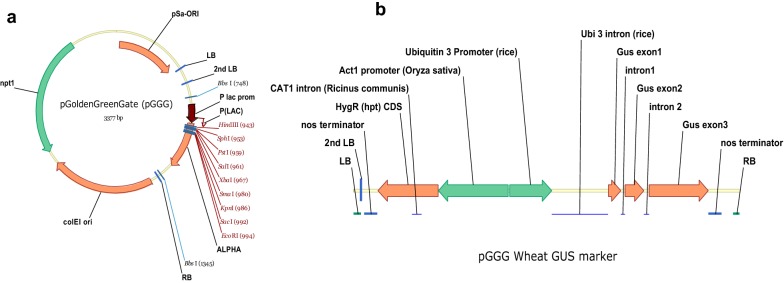
**a** The pGoldenGreenGate-M (pGGG-M) a GoldenGate (MoClo) level 2 vector based on pGreen. **b** pGGG containing the rice actin promoter driving the hygromycin (*hpt*) selection gene containing the CAT1 intron and the rice ubiquitin promoter driving the GUS maker gene containing two introns

To create a pGreen [18] based, Golden Gate cloneable vector compatible with the Modular Cloning (MoClo) system a 799 bp fragment containing the LB and a LacZ Golden Gate cassette was isolated from pAGM8031 (Addgene 48037) using restriction enzymes *Psh*AI/*Pme*I, and cloned into pGreen II 0000 at the *Hpa*I/*Stu*I sites using blunt end ligation. This Level 2 binary vector was deemed pGoldenGreenGate-M (pGGG-M) (Fig. 2a). A reporter pGGG vector was made, for wheat transformation, which contained the hygromycin resistance gene (*Hpt*) and *Cat*1 intron driven by the rice actin1 promoter, and the β-glucuronidase gene with 2 introns (*GUS*2Int) driven by the rice ubiquitin promoter (Fig. 2b). Briefly, the Level 1 constructs pICH47802-RActpro::HptInt::NosT (selectable maker) and pICH47742-RUbipro::GUS2int::NosT (GUS Reporter) were cloned into the binary Level 2 vector pGGG-M using standard Golden Gate MoClo assembly [19].

### Preparation of *Agrobacterium* for transformation

The hypervirulent *Agrobacterium tumefaciens* strain AGL1 [20] was used in all plant transformation experiments. Vectors were electroporated into *Agrobacterium* AGL1 competent cells as previously described [2], when pGreenII [18] derivatives were used i.e. pBRACT [21] or pGGG they were co-electroporated with the helper plasmid pSoup [18] or its derivatives pAL154 contained the 15 kb Komari fragment or pAL155 with an additional *Vir*G gene.

Single colonies of *Agrobacterium* AGL1, which contain the desired vector, were inoculated into 10 mL of LB [22] liquid medium containing appropriate antibiotics and incubated at 28 °C, shaken at 200 rpm for ~ 65 h. A modified method of Tingay et al. [23] to prepare *Agrobacterium* standard inoculums for transformation was used as previously described by Bartlett et al. [2]. Equal quantities of 30% sterile glycerol and the *Agrobacterium* culture were mixed by inverting and aliquots of 400 μL in 0.5 mL Eppendorf tubes were made. The aliquots of standard inoculums were frozen at − 80 °C and stored until required.

The day before wheat transformation a single 400 μL standard inoculum was used to inoculate 10 mL of liquid MG/L [24] (5 g L^−1^ mannitol, 5 g L^−1^ tryptone, 2.5 g L^−1^ yeast, 100 mg L^−1^ NaCl, 1 g L^−1^ Glutamic acid, 250 mg L^−1^ KH_2_PO_4_, 100 mg L^−1^ MgSO_4_, 1 μg L^−1^ Biotin. final pH = 7) medium without antibiotics and incubated at 28 °C shaken at 200 rpm overnight (~ 16 h). On the day of transformation, the bacteria were pelleted by centrifugation in a 50 mL Falcon tube at 3100 rpm for 10 min at 24 °C. The supernatant was discarded, and the cells resuspended gently in 10 mL wheat inoculation medium (WIM) to an optical density of 0.5 OD (600 nm) and 100 µM AS added. The culture was incubated at room temperature with gentle agitation (80 rpm) for 4–6 h in the dark.

### Inoculation with *Agrobacterium* and co-cultivation

The isolated embryos were placed into fresh WIM medium prior to centrifugation at 14,000 rpm at 4 °C for 10 min [25]. WIM was removed with a pipette and after 1 mL *Agrobacterium* solution added, the tubes were inverted frequently for 30 s and incubated at room temperature for at least 20 min. After the incubation period, the *Agrobacterium* suspension was poured with the embryos into a 50-mm diameter Petri plate and the *Agrobacterium* suspension was removed with a pipette. The embryos were transferred, scutellum side up, to the co-cultivation medium which consisted of WIM supplemented with 100 μM AS, 5 µM AgNO_3_, 1.25 mg L^−1^ CuSO_4_·5H_2_O and 8 g L^−1^ agarose [11]. Twenty-five embryos were placed in each 90 mm single vent Petri plate (Thermo Scientific No 101R20) and incubated at 24 ± 1 °C in the dark for 3 days co-cultivation (Fig. 1f).

Throughout the tissue culture process, all solid media components, except for the gelling agent, were prepared as a double-concentrate and filter-sterilised. The gelling agents were prepared as a double concentrate in water and sterilised by autoclave. After autoclaving the gelling agents (2×) were maintained at 60 °C and the filter-sterilised media components (2×) were warmed to 60 °C prior to mixing both and pouring. The phytohormones and antibiotics were added as filter sterilised stocks just before pouring.

### Resting period, callus induction and selection of transformed material

After 3 days’ co-cultivation, the embryogenic axes were excised from the embryos using forceps (Fig. 1g). The embryos were transferred to the fresh callus induction plates (WCI) based on the media described in [26] but containing 2 mg L^−1^ Picloram (Sigma-P5575), 0.5 mg L^−1^ 2,4-dichlorophenoxyacetic acid (2,4-D), 160 mg L^−1^ Timentin and 5 mg L^−1^ agarose and incubated at 24 ± 1 °C in the dark for 5 days. Timentin was added to control *Agrobacterium* during the resting period. The embryos were transferred, scutellum side up, to fresh WCI plates as above with 15 mg mL^−1^ Hygromycin and incubated at 24 ± 1 °C in the dark for 2 weeks. This transfer is referred to as Selection 1. The calli were split at the next transfer into clumps of approximately 4 mm^−2^, callus pieces derived from each single embryo were labelled to keep track of their origin. The calli were transferred to fresh selection plates (WCI) as above, but with 30 mg L^−1^ Hygromycin (Selection 2) and incubated at 24 ± 1 °C in the dark for 2 weeks (Fig. 1h). The number of explants per plate were reduced by approximately half at Selection 2. After 2 weeks the calli were transferred to a lit culture room under fluorescent lights (100 μmol m^−2^ s^−1^) at 24 ± 1 °C with a 16-h photoperiod and covered with a single layer of paper towel for a further week. During this period putative transformed lines should start to green and produce small shoots (Fig. 1i).

### Regeneration of transgenic plants

After the 3 weeks on Selection 2 medium, the calli were transfer one final time to wheat regeneration medium (WRM) containing 4.4 g L^−1^ MS (Duchefa M0222), 20 mg L^−1^ sucrose, 0.5 mg L^−1^ MES supplemented with 0.5 mg L^−1^ Zeatin, 160 mg L^−1^ Timentin and 20 mg L^−1^ Hygromycin, 3 g L^−1^ Gelzan (Sigma-Aldrich) in deep Petri dishes (tissue culture dish, 90 mm diameter × 20 mm, Falcon 353003). All regenerating callus derived from a single embryo was labelled to track its origin. The paper covering was removed and the calli were cultured under fluorescent lights (100 μmol m^−2^ s^−1^) at 24 ± 1 °C with a 16-h photoperiod.

### Rooting

Regenerated shoots which were 1–2 cm in length with visible roots (Fig. 1j) were transferred to “De Wit” culture tubes (Duchefa, W1607) containing 8 mL of WCI without growth regulators, solidified with 3 g L^−1^ Gelzan and supplemented with 160 mg L^−1^ Timentin and 15 mg L^−1^ Hygromycin. A strong root system with root hairs developed on putative transformed plants (Fig. 1k).

### Acclimatisation

Regenerated plantlets with strong root systems (Fig. 1l) were gently removed from the tubes using long forceps and the roots gently washed with cool running water to remove any remaining tissue culture medium. They were planted in a peat and sand mix in 5 cm square cell trays and covered with a clear plastic propagator lid. To maintain high humidity around the plants, they remained covered with the propagator lids for approximately 1 week while they became established in soil. Within a controlled environment room, the plants were grown at 18 ± 1 °C during the day (16 h) and 15 ± 1 °C at night temperatures, with relative humidity maintained at 65%, metal halide lamps (HQI) supplemented with tungsten bulbs provided a light intensity of with 400–600 μmol m^−2^ s^−1^ a 16 h photoperiod.

### GUS histochemical assay

The GUS activity was determined after co-cultivation, resting, Selection 1, after rooting medium and on T1 seed in the next generation using a GUS histochemical assay. The plants were immersed in GUS assay substrate containing 1 mmol L^−1^ of 5-bromo-4-chloro-3-indolyl glucuronide (X-gluc), 100 mmol L^−1^ sodium phosphate, 10 mmol L^−1^ Na_2_EDTA and 0.1% of triton X-100, pH = 7 at 37 °C under dark conditions overnight (~ 16 h). All green samples were decoloured and fixed in 70% ethanol to remove chlorophyll and other plant pigments prior to visualising and photographing.

### DNA extraction

0.5 to 0.7 cm leaf samples were harvested in PCR tubes, and DNA was extracted by Extract-N-Amp™ Plant Tissue PCR Kits (Cat No. XNAP-1KT) following the manufacturer’s instructions.

### HygR (*hpt*) polymerase chain reaction (PCR)

A 335 bp amplicon of the hygromycin *hpt* gene was PCR amplified using the primer pair HygF 5′-AGGCTCTCGATGAGCTGATGCTTT-3′, Hyg Reverse 5′-AGCTGCATCATCGAAATTGCCGTC-3′ and REDExtract-N-Amp PCR Reaction Mix (Cat No. XNAS) with a 20 µL total volume per reaction. Each reaction comprised of 10 µL PCR Reaction Mix (REDExtract-N-Amp), ~ 50 ng of plant genomic DNA, 1 µL (10 mM) of each primers (Hyg F and Hyg R), and sterile laboratory grade water up to a total volume of 20 µL. PCR was performed in a Peltier Thermal Cycler 200 (MJ Research), with the conditions 95 °C for 3 min, followed by 34 cycles of 95 °C for 30 s, 58 °C for 30 s, 72 °C for 1 min, then 72 °C for 7 min before a final hold of 10 °C. PCR products were resolved by gel electrophoresis on a 1% agarose gel which contained ethidium bromide at 1 μg 10 mL^−1^.

### Quantitative real-time PCR to determine transgene copy number

Approximately 100 mg leaf samples were placed into 1.5 mL Eppendorf tubes and using liquid nitrogen flash frozen. The leaf material was stored at − 80 °C if DNA extraction could not be performed immediately. DNA was extracted from the leaf material using the Qiagen DNeasy plant mini kit (Cat No. 69106) according to the manufacturer’s instructions. A Nanodrop ND-1000 spectrophotometer was used to assess DNA concentrations.

iDna Genetics performed Quantitative real-time PCR using the hygromycin resistance gene (*hpt*) and *CO2* (Constans-like, AF490469) gene specific probes and primers as described in Bartlett et al. [2]. Using the design module “TaqMan Probe and Primer” of the Applied Biosystems software Primer Express, target sequence specific primers were designed. The reactions used low rox version of the Absolute mix (Catalogue AB1318B, ThermoScientific). Multiplex assays were performed on the *hpt* gene and the *CO2* gene. The final concentrations of probes and primers were at 200 nM. Each assay contained 5 μL of DNA solution, which was optimised for final DNA concentrations 1.25 to 10 ng μL^−1^ (6.25 to 50 ng DNA in each assay). PCRs were performed in an Applied Biosystems Quantstudio5 Machine equipped with a 384-place plate. The PCR cycling conditions were 95 °C 15 min (activation of enzyme), 40 cycles of 95 °C 15 s, 60 °C 60 s.

## Results

### Optimization of wheat transformation with *Agrobacterium*

#### Effect of gelling agent on callus induction and regeneration

An initial study was performed to examine the regeneration capacity of wheat IEs, without an *Agrobacterium* treatment, 40 embryos were cultured per gelling agent with four different gelling agents (2% Gelzan G1910, 5% Agarose A9045, 3.5% Phytagel P8169 Sigma-Aldrich, and 8% Agar AGA03 For Medium) to identify which gelling agent yielded the highest percentage of regenerated plants.

**Fig. 3:**
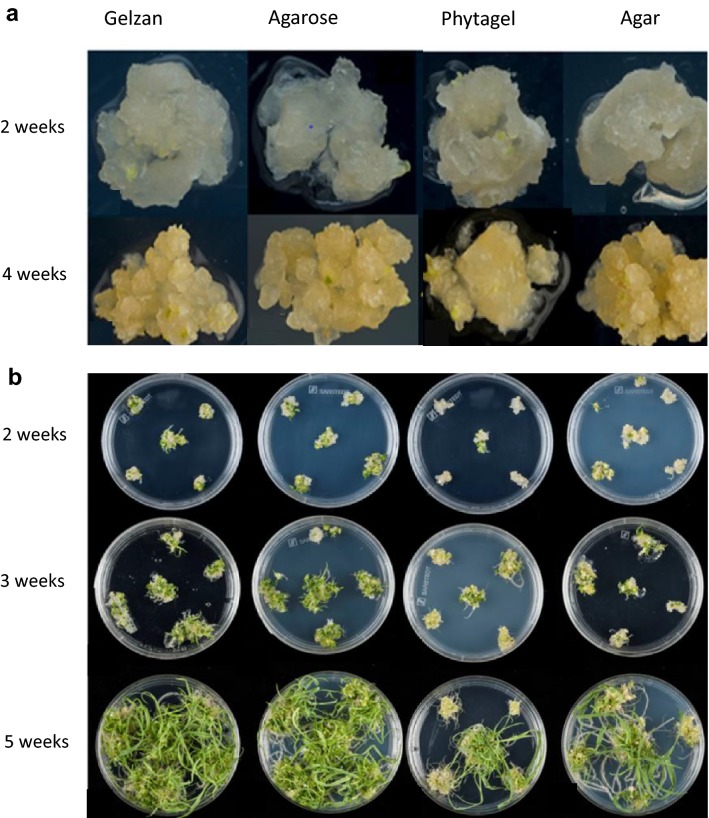
**a** The effect of gelling agent on callus formation on WCI with Gelzan, Agarose, Phytagel and Agar, maintained in the dark. **b** The effect of gelling agent on shoot regeneration from wheat calli when cultured on WRM with Gelzan, Agarose, Phytagel and Agar, maintained under 16 h light

Throughout the 5-week callus induction phase of this experiment there was little difference visible to the naked eye in callus development on each type of gelling agent. However, when examined under the microscope, embryo-derived callus on Agarose or Gelzan appeared to have a better embryogenic structure (Fig. 3a).

The calli were transferred to WRM with the four different gelling agents. A notable difference in the performance of the gelling agents was observed after 4 weeks on WRM. The majority of shoots that regenerated on Agar and Phytagel remained stunted for the remainder of regeneration stage; whilst the shoots on Gelzan, and Agarose had extensive shoot development reaching in excess of 10 cm in length (Fig. 3b). The gelling agents performance in terms of the percentage of IE-derived calli to produce shoots larger than 1 cm was Gelzan 81%, Agrose 77.5%, Phytagel 51.5% and Agar 57.5%. For all further experiments we chose to use Agarose for callus induction and Gelzan for regeneration and rooting.

#### Pre-treatment and co-cultivation

To examine the effect of the centrifugation pre-treatment, 150 IE were isolated and placed in Eppendorf tubes containing WIM, half the embryos (75) were subjected to centrifugation at 14,000 rpm at 4 °C for 10 min. The controls without centrifugation were incubated at 4 °C for 10 min. Both centrifuged and control treatments had the WIM removed and were then inoculated with *Agrobacterium* AGL1 containing pGGG as previously described in the methods section. The IE’s were then co-cultivated for 3 days. Subsets of 10 embryos/embryo derived calli were sacrificed for GUS staining at two stages; after the resting period and after the first selection stage. In the centrifuged group, 90% of the embryos and 60% of the calli chosen for GUS staining showed blue foci, while in the control group, only 50% of embryos and 50% of calli were GUS positive. The degree of the blue foci after resting and first selection was stronger in the centrifuged group than the untreated control. Between groups the difference was statistically significant (P < 0.05) after the resting period. The remaining calli, 55 in each treatment, were taken through the entire transformation process. The number of individual embryos yielding calli that regenerated shoots was higher in the pre-treated centrifuged group when compared to the untreated control group. Plants were regenerated from 15 calli out of 55 (27.3%) that were derived from the embryos pre-treated by centrifugation, while only three embryos derived calli regenerated plants (5.5%) from the control group. Ten of the regenerated plants expressed GUS in the centrifuged group giving a transformation efficiency of 18.2%. Of the untreated control group only one plant expressed GUS, giving 1.8% transformation efficiency. Therefore, centrifugation at 14,000 rpm at 4 °C for 10 min was used in our all transformation experiments.

#### Effect of embryo orientation during culture

Immature embryos were cultured with their scutellum side either in contact with the medium or facing upwards during and after their co-cultivation with *Agrobacterium* AGL1 containing pGGG. The differences between the two groups in terms of GUS staining were tested after co-cultivation, a resting period and on the first selection medium. One hundred embryos were used as starting material in each group and 25 embryos/calli per group were tested for GUS expression after each stage. The staining was scored under a light microscope. Each individual embryo/callus was given a score. The increasing number of the score was an indicator of strength (degree) of the staining. Scoring started from 0 (no GUS expression seen), score 1 (1–5 foci ~ quarter of the material), score 2 (5–10 foci ~ half the material) score 3 (10–15 ~ three quarters of the material) and score 4 (almost totally stained). The statistical analyses showing the transformation efficiency differences between the groups were performed using unpaired *t* test in Genstat 18th edition software. A *P*-value less than 0.05 was considered to be statistically significant.

**Fig. 4:**
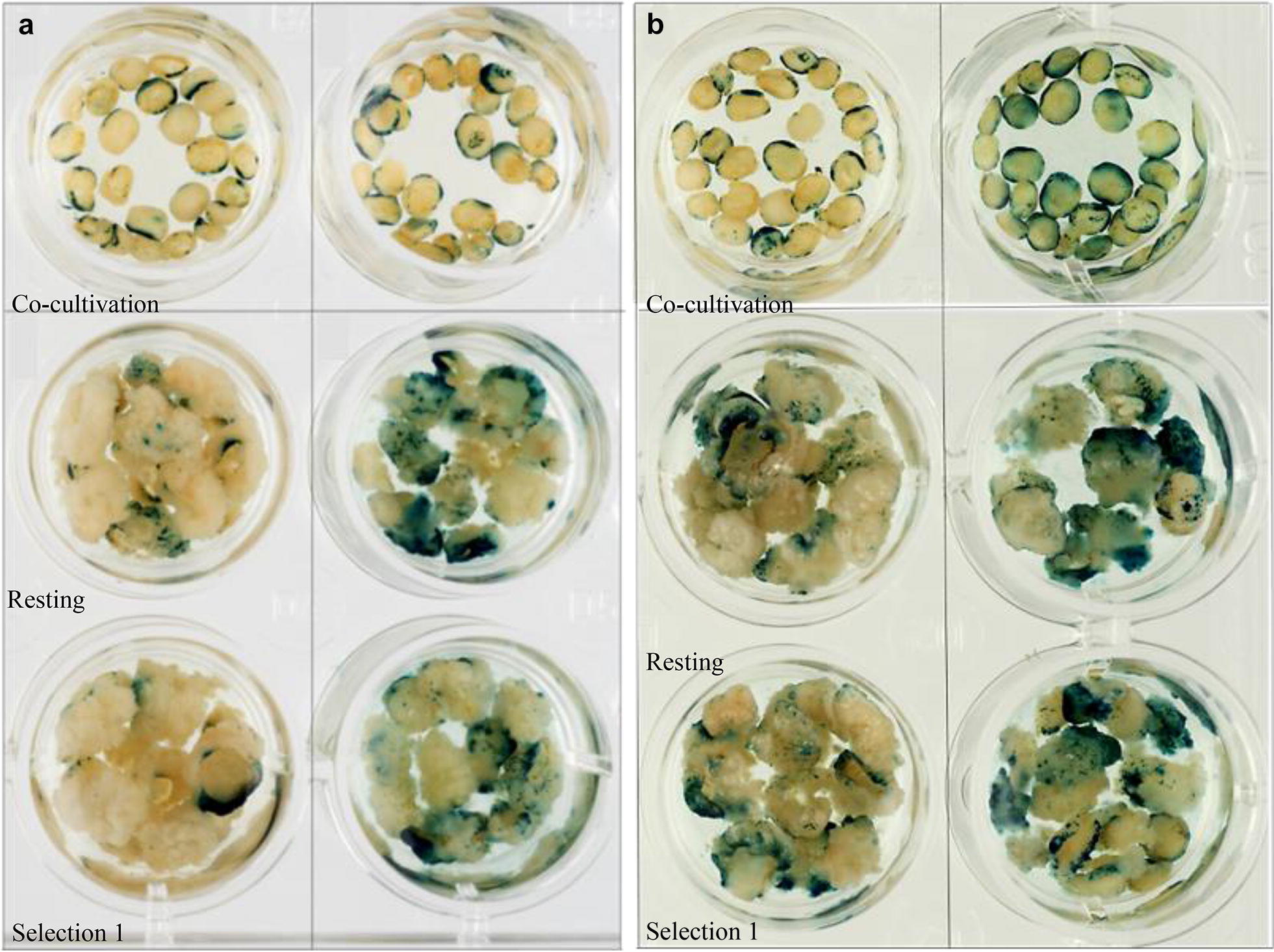
**a** GUS staining images of scutellum down (the first column) and up (the second column) groups after co-cultivation/resting/selection 1. **b** GUS staining images of co-cultivation 2 days (the first column) and 3 days (the second column) groups after co-cultivation/resting/selection 1

Based on transient expression of the *GUS* gene, embryos co-cultured scutellum side up had more staining than those cultured scutellum side down. Although 100% of the embryos tested were stained blue in both groups, scutellum side up co-cultured embryos showed blue foci on both embryo sides, whereas embryos co-cultured scutellum side down had mainly GUS staining on the axis side periphery. Similarly, after the resting period and first selection, calli from embryos cultured scutellum side up had more blue foci than those cultured scutellum side down and GUS staining was stronger (Fig. 4a). The difference between groups was significant (P < 0.05) after cultivation on Selection 1 medium according to the scoring system of GUS stained embryos mentioned above. IEs were cultured scutellum side up throughout the transformation procedure in all subsequent experiments.

#### Length of co-cultivation

The effect of co-cultivation period (2 days or 3 days) was investigated; embryos were isolated and inoculated on the same day, then transferred to resting medium either after 2 days or 3 days co-cultivation. GUS staining was performed after the resting period and after the first selection. Twenty-five embryos/calli per group were checked for their GUS expression (Fig. 4b). Although 100% of the embryos/calli stained blue in both groups, embryos co-cultured for 3 days had stronger GUS staining than those co-cultured for 2 days. Using the same scoring system, the difference between groups was statistically significant (P < 0.05) after resting. We used 3 days co-cultivation time in all further experiments.

#### Transformation efficiency, selection and plasmid backbone

**Fig. 5:**
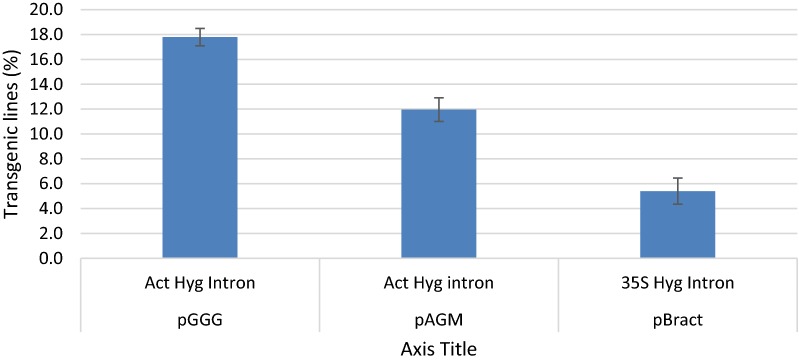
Transformation efficiencies as percentages for the three constructs used in this study pGGG, pAGM and pBRACT

**Fig. 6:**
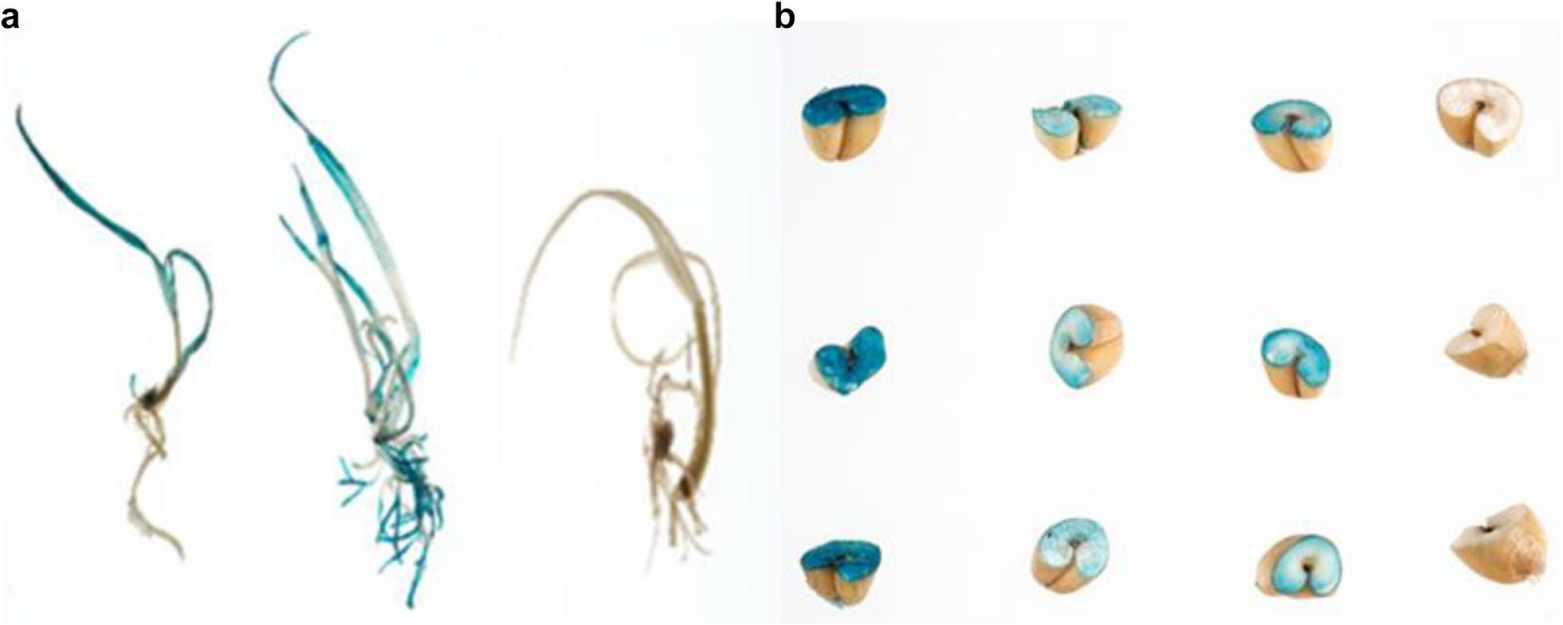
**a** Transgenic plants showing GUS expression after being on rooting medium and Fielder (non-transformed) tissue culture control on the right. **b** Segregating T_1_ generation seeds showing GUS expression in three independent transgenic lines

The improved protocol, as reported earlier in the methods section, was applied to 3289 IEs over 27 experiments, which gave rise to 380 transformed plants, our transformation efficiencies ranged from 5 to 25% (Table 1). Significant differences in transformation efficiencies were found between the three constructs using pGGG (18%), pAGM (12%) and pBRACT (5%) (P < 0.05) (Fig. 5). Furthermore, significant differences in transformation efficiencies were seen when different promoters were used (CaMV 35S or rice actin) to drive the hygromycin (*Hpt*) resistance gene containing the CAT1 intron (P < 0.001). Actin:*hpt* in either pGGG or pAGM backbones outperformed the 35S:*hpt* selection in pBRACT (Fig. 5). The plasmid backbone containing the actin:*hpt* selection was found to have a significant effect on transformation efficiency (P < 0.001). The pGGG vector gave a higher average transformation efficiency (18.22%) when compared to pAGM8031 (12.03%), both constructs contained an identical actin:*hpt* selection cassette. Lines created with the pBRACT construct, containing the 35s:*hpt*, gave the greatest number of escapes (non-transformed lines) coming through the transformation process, out of 93 regenerated plantlets, 11 were escapes (11.8%), when compared to actin:*hpt* selection at 133 plantlets, 8 escapes (6%). To help eliminated escapes, it was observed that if plants produced strong roots which showed root hairs in media containing hygromycin selection, they were usually found to be transformed, whereas, those without root hairs were escapes, later confirmed by PCR and/or GUS staining (Fig. 6a). Figure 6b shows *gus* gene expression in T1 seeds showing segregation in the next generation.

**Table 1:** Effects of promoter driving hygromycin selection and plasmid backbone on transformation efficiency

BackBone	Promoter driving the selectable marker gene	Number of inoculated IE	Number of independent transgenic plants	Transformation efficiency (%)
pGGG	Act Hyg Intron	150	14	9
pGGG	Act Hyg Intron	175	27	15
pGGG	Act Hyg Intron	160	40	25
pGGG	Act Hyg Intron	100	18	18
pGGG	Act Hyg Intron	25	5	20
pGGG	Act Hyg Intron	25	6	24
pGGG	Act Hyg Intron	80	19	24
pGGG	Act Hyg Intron	175	12	7
pAGM	Act Hyg Intron	100	15	15
pAGM	Act Hyg Intron	100	12	12
pAGM	Act Hyg Intron	100	5	5
pAGM	Act Hyg Intron	100	14	14
pAGM	Act Hyg Intron	100	24	24
pAGM	Act Hyg Intron	200	14	7
pAGM	Act Hyg Intron	125	21	17
pAGM	Act Hyg Intron	150	13	9
pAGM	Act Hyg Intron	150	16	11
pAGM	Act Hyg Intron	80	18	23
pAGM	Act Hyg Intron	150	11	7
pAGM	Act Hyg Intron	125	11	9
pAGM	Act Hyg Intron	150	15	10
pAGM	Act Hyg Intron	150	10	7
pAGM	Act Hyg Intron	100	11	11
pBract	35S Hyg Intron	80	4	5
pBract	35S Hyg Intron	150	10	7
pBract	35S Hyg Intron	100	5	5
pBract	35S Hyg Intron	100	5	5
pBract	35S Hyg Intron	100	5	5
pBract	35S-Hyg-Intron	39	2	5
pBract	35S-Hyg-Intron	50	3	6

#### Copy number results

**Fig. 7:**
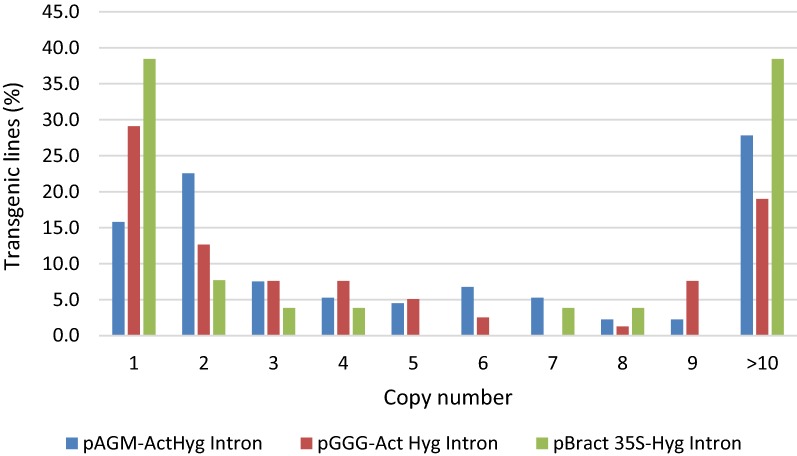
Comparison of copy number data using the different plasmid backbones and selection cassettes

The copy number data revealed differences between each of the constructs used. The highest percentage of single T-DNA insertion lines were created using the pBRACT construct, with 38.5% of lines being single copy, pGGG gave rise to 29.1% single copy lines. The pAGM construct gave the lowest percentage of single wheat copy lines at 15.8% (Fig. 7). Wheat lines created using the pAGM construct gave rise to more two copy lines (22.6%) than lines with a single copy, furthermore, 27.8% of lines created using this construct had high copy number, containing more than 10 transgene copies (Fig. 7). The pGGG and pBRACT constructs produced 19% and 38.5% respectively, of high copy lines with more than 10 copies of the transgene (Fig. 7).

Transgene copy number analysis was performed on multiple individual plants arising from the same single embryos in a subset consisting of 24 embryonic lines. The copy number differed between plants taken from the same embryo in 19 of the 24 lines tested (79.2%). This suggests that these are independent transformation events and that the occurrence of multiple independent events from single embryos is common.

Initial studies were performed to assess the effect, on transformation efficiency, of including additional virulence (*vir*) genes on pSOUP derivatives within *Agrobacterium* AGL1. The GUS reporter pGGG construct was included in AGL1 along with either the standard pSOUP plasmid, or its derivatives pAL154 contained the 15.2 kb Komari fragment, or pAL155 with an additional *virG*^542^ gene. Isolated IEs were treated as previously described in the method section and sacrificed for GUS staining 5 days after *Agrobacterium* inoculation. Twenty-four embryos were used per treatment and experiments repeated twice. Differences were not seen between the embryos treated with AGL1 containing pGGG plus the standard pSOUP and AGL1 containing pGGG plus pAL155 *virG*^542^, 94.4% and 95.83% respectively. However, embryos treated with AGL1 containing pGGG plus pAL154 showed GUS staining in 54.16% of embryos.

#### Transgene inheritance and segregation

All transgenic plants produce in this study had a normal phenotype and set seed. To prove germline inheritance of the *GUS* transgene GUS analysis was performed on dried mature T1 seed. Three single copy transgenic T1 lines (copy number determined by qPCR) were chosen. 48 seed from each T1 line were randomly selected, cut in half and stained for GUS as described in “Materials and methods” section. Non-transformed Fielder seed were used as a negative control. Two of the lines tested each had 33 seeds which were GUS positive (blue) and 15 GUS negative (white) null segregants, the remaining line had 37 GUS positive seed and 11 white negative null segregants. From the 144 seeds tested, if following a Mendelian inheritance ratio of 3:1, one would expect 108 GUS positive and 36 GUS negative null segregants. The observed 103 GUS positive and 41 GUS negative null segregants is not significantly different to the expected (*X*^2^ P = 0.505602 not significant at P < 0.05), and therefore follows a 3:1 inheritance ratio.

## Discussion

**Fig. 8:**
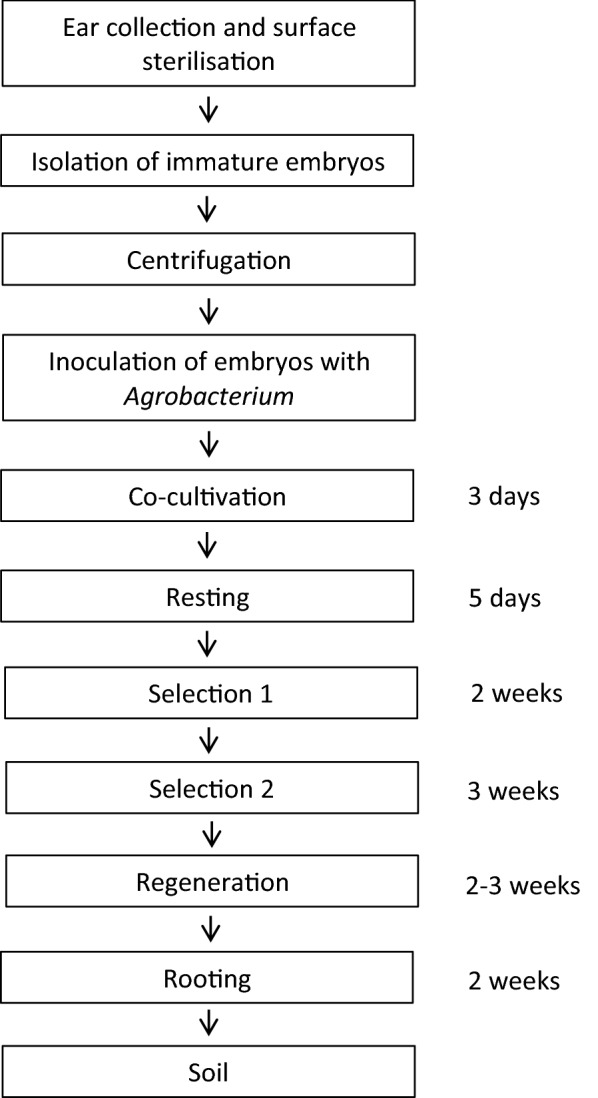
Timeline showing the main steps of *Agrobacterium*-mediated transformation in wheat

Plant transformation technologies which enable genetic modification are invaluable tools for functional genomic studies and crop improvement programmes [21]. Transformation efficiency for wheat has languished around 5% for many years despite its global importance [14]. In the present study we have developed an efficient, reproducible and transferrable transformation method for wheat. The transformation process takes approximately 11 weeks (Fig. 8) and has been used successfully over several years. We report some of the key factors influencing wheat transformation efficiency and provide a detailed reproducible protocol.

Conveniently, stable plant transformation can be divided into two components: the phases that enable DNA transfer to the cell and integration into the genome effectively, and those which enable the selection and regeneration from transformed cells of whole viable plants [27]. The driving force behind improvements to transformation systems are the increased demands for systems that are high-throughput, highly efficient and cost-effective [9]. These improvements are partly due to the development of improved tools for gene delivery and genetic manipulation and through tissue culture regime refinement enabling better plant regeneration. DNA cloning approaches and the technologies used to implement them, underpin and are often the starting point for most gene function studies [21].

In our experience, a key feature essential for success is good quality, heathy, donor material grown under controlled environmental conditions. Donor plants should not be sprayed with pesticide at any growth stage, therefore, good plant hygiene practices need to be in place. The conditions of the controlled growth chamber should be adjusted to produce vigorous growing plant material so that the IEs reach the early milk stage GS73, approximately, 14 days post anthesis. Collection of IEs that are healthy and at the correct stage is key.

### Pre-treatment and co-cultivation

Pre-treatment by centrifugation, embryo orientation during co-cultivation with *Agrobacterium* and the duration of co-cultivation were all important factors affecting DNA delivery and transformation efficiency in wheat. Silwet L-77 usage and centrifuge pre-treatment were already proven to increase the wheat transformation efficiency of IEs [8, 11, 28]. Pre-treatment by centrifugation is assumed to increase the cell wall membrane permeability and the penetrance of *Agrobacterium*, resulting in improved DNA delivery and transformation efficiency. However, the mechanism by which the centrifugation pre-treatment affects transformation is still unknown.

We examined whether the orientation of the immature wheat embryos during culture affected expression of the GUS reporter gene. Traditionally, wheat embryos are cultured scutellum upward, as are maize embryos, within the transformation process, whereas, barley embryos are cultured scutellum down, with the scutella in contact with the medium surface [29]. In the first reported *Agrobacterium*-mediated transformation of wheat, by Cheng et al. [8], embryos were cultured scutellum up. We found that embryos cultured scutellum side up gave stronger GUS staining after the resting period and the first selection.

The majority of wheat transformation protocols ambiguously state that co-cultivation of embryos with *Agrobacterium* is performed for 2 to 3 days [8, 14, 30–32]. Although, some are specific, 2 days [11, 33] or 3 days [34]. In this study, under the reported conditions, we found that 3 days co-cultivation had significantly stronger GUS expression when tested after the resting period, however, after the first selection the difference was not as pronounced. This might suggest an increased in T-DNA delivery and transient gene expression, but DNA integration and stable gene expression may be similar in both treatments.

### Gelling agent

The main constraint for enhancing *Agrobacterium*-mediated wheat transformation is the capacity to effectively regenerate transgenic plants from transformed callus. We investigated the effect of different gelling agents (Gelzan, Agarose, Phytagel and Agar) on the regeneration from IEs of the wheat cultivar Fielder over a 9-week culture period. The purpose was to identify which gelling agent yielded the highest quantity of shoots larger than 1 cm in length and the highest proportion of regenerating embryos.

In terms of regeneration frequency throughout the two experiments, Gelzan (81%) and Agarose (77.5%) were the most consistent at producing shoots > 1 cm and produced the highest average number of shoots. Phytagel and Agar were consistently poor regarding regeneration frequency.

The influence of five different gelling agents has also been reported by Berrios et al. [35], looking at Phytagar, Phytagel, Agarose, Arcagel and Agar–Agar. They used multiple recombinant inbred lines of sunflower cotyledons. Their findings similarly reported significant differences depending on the gelling agent used. Agarose induced positive regeneration by organogenesis.

### Effect of selection cassette and plasmid backbone

In the present study, the first requirement was a binary vector that allowed efficient and straightforward wheat transformation. We required the ease and speed of the modular cloning (MoClo) system based on type IIS restriction enzyme cloning “Golden Gate assembly” as described by [19]. Furthermore, we required an easy method for including additional virulence genes within the strain of *Agrobacterium* as several previous publications report the importance of additional virulence genes within wheat transformation experiments [14, 36]. Hence, we developed the MoClo compatible pGoldenGreenGate (pGGG) vector based on pGreen and therefore, compatible with the helper plasmid pSoup and its derivatives containing additional virulence genes [18].

The pBRACT vector, also used in this study, contains the 35S promoter driving an intron enhanced hygromycin resistance gene and is highly efficient at transforming barley [2, 21]. The CaMV 35S promoter does express in monocotyledonous plants, but its comparative strength is considerably lower in monocotyledonous than in dicotyledonous plant cells [37]. Transgene expression in transgenic plants can be strongly enhanced by the inclusion of introns in many cases [38]. The inclusion of a castor bean catalase-1 (CAT-1) intron within the *Hpt* gene has been shown to increase transgene expression by approximately 2.5 fold and improved transformation efficiency in rice and barley [39, 40]. The use of monocot derived promoters to drive transgenes within monocot transgenic plants has resulted in a high degree of gene expression [37]. Our results concur with Jang et al. [37], in that, in wheat, the 35S promoter within pBRACT was successively out performed by the monocot derived rice actin (*OsAct*1) promoter when driving the intron enhanced *Hpt* gene within pGGG or pAGM.

### Agrobacterium tumefaciens strains and binary vectors

An *Agrobacterium* strains ability to transform plant cells is determined by its plasmid and chromosomal genomes, all the machinery necessary for cell attachment and DNA-transfer is encoded by them [14]. There are only two chromosomal backgrounds that have been used successfully to transform wheat, one is Ach5 of strain LBA4404 [41] the other is the C58 background, both have been used with a variety of Ti plasmids and binary vectors. An important group of C58 strains that have played key roles in wheat transformation are EHA101, EHA105, AGL0, and AGL1 [20, 42, 43], originating from strain A281 that contains the hypervirulent pTiBo542 Ti plasmid which harbours additional *vir* genes. The introduction to *Agrobacterium* of *vir* gene copies or combinations, has been achieved by using alternative Ti plasmids, additional helper plasmids or on the backbone of binary vectors containing additional virulence genes [44]. The ability of *Agrobacterium* strains containing the hypervirulent Ti plasmid pTiBo542 to confer higher transformation efficiencies in wheat have been demonstrated in several comparative studies [32, 45]. The present study utilises the hypervirulent *Agrobacterium* strain AGL1 containing pTiBo542. Transformation of wheat with *Agrobacterium* LBA4404, a weakly virulent strain, was only successful when a superbinary vector was used that contained additional *vir* B, C and G genes from pTiBo542 [46]. The beneficial effect of extra *vir* genes was illustrated by increased T-DNA transmission and transformation of wheat when the 15 Kb Komari fragment from pTiBo542 was included on the pSoup helper plasmid in the hypervirulent strain AGL1 [47]. Wang et al. [48] found that transformation efficiencies doubled, to 4%, with the addition of the Komari fragment or an additional *vir* gene regulator *vir*G. In our initial transient expression studies, the inclusion of the Komari fragment (*virB*/*C*/*G*) on the pSoup derivative pAL154 was detrimental to T-DNA transfer, yielding 54% GUS stained embryos compared to the control pSoup or the pSoup derivative pAL155 with the additional *virG*^542^, both yielding ~ 95% of embryos displaying GUS staining. The addition of pAL154 (*virB/C/G*) with pGGG within the *Agrobacterium* may have caused an imbalance in the transformation machinery. In stable transformation experiments, that compared the standard pSoup plasmid and pAL155 *virG*^542^, a slight, non-statistically significant, improvement was seen in transformation efficiency when the additional *virG*^542^ was used, 17 ± 3.2% compared to 19 ± 4%. The high ~ 95% of embryos showing GUS expression in our transient studies would suggest that future further improvement in transformation efficiencies will be achieved through improvement in plant regeneration rather than with improved DNA transfer. The incorporation of additional *vir* genes into binary vectors is not always necessary, a substantial amount of transgenic lines using normal *Agrobacterium* strains and binary vectors have been documented [14].

In experiments comparing the vector backbones, we found that pGGG had a higher transformation efficiency than pAGM, 18% compared to 12% respectively. We also found that pGGG gave a higher percentage of transgenic lines containing single copy T-DNAs compared to pAGM, 29.1% compared to 15.8%. In bacteria, the plasmid origin of replication determines the number of plasmid copies within a bacterial cell. Binary plasmids contain two origins of replication, one enables replication within *E. coli*, the second, “broad-spectrum” origin of replication, allows replication within *Agrobacterium*. The broad-spectrum replication origin contained on pGGG is pSa ori and on pAGM it is pVS1 ori, plasmids with these origins are maintained at around 4 copies per cell and 7–10 copies per cell, respectively [49, 50]. The additional copies of pAGM within *Agrobacterium*, in comparison to pGGG, may offer some insight into explaining the higher T-DNA copies found integrating in transgenic lines transformed with pAGM. A restricted number of T-strands transmitted to the plant cell may lead to low incorporation and therefore lower transgene copy numbers in plants. A correlation between plant and bacterial T-DNA copy number, may be expected, when transforming with different plasmids containing different origins of replication, as they replicate to varying extents within the *Agrobacteria* [50]. T-DNA copy number may also be influenced by the strain of *Agrobacterium*, method of transformation and target tissue used [51]. Multiple interactions at many levels occur during the transformation process, interactions between the plant material, bacterial cell, bacterial chromosome, Ti plasmid and binary vector.

We have demonstrated that the GUS gene is stably transmitted to the next generation and is expressed in T_1_ seeds (Fig. 6b). A Mendelian inheritance ratio of 3:1 was observed in transgene transmission, as one would expect from single copy T-DNA transgenic lines.

The transformation efficiency throughout the paper was calculated as single events occurring from each embryogenic line (1 plant from 1 embryo) and displayed as the percentage of positive transgenic plants produced from the total number of IEs isolated and inoculated with *Agrobacterium* in an experiment. However, copy number data showed that plants derived from a single embryonic line had different copy numbers, 19 of the 24 lines tested (79.2%). This suggests that multiple transformation events, from single embryogenic lines, occur often and our transformation efficiency might be much higher than we have calculated.

## Conclusion

Developing enhanced functional wheat genomics tools is of the utmost importance for the wheat scientific community and breeders. Here we report a high-throughput, highly efficient and repeatable transformation protocol for the wheat cv ‘Fielder’. The protocol has been successfully transferred to other research groups. Work is underway to further test this protocol in other wheat cultivars. New technologies such as genome editing, using CRISPR–Cas systems rely heavily on efficient, reproducible transformation systems. Our method has already been used extensively for both gene characterisation studies and genome editing in wheat. It is extremely probable that even greater efficiencies will be accomplished with further optimisation, allowing more rapid exploitation of new genomic resources and genome editing technologies.

## Data Availability

The datasets supporting the conclusions are included within the article.
