# Correction to: An efficient and reproducible *Agrobacterium*- mediated transformation method for hexaploid wheat (*Triticum aestivum* L.)

**DOI:** 10.1186/s13007-019-0540-7

**Published:** 2019-12-17

**Authors:** Sadiye Hayta, Mark A. Smedley, Selcen U. Demir, Robert Blundell, Alison Hinchliffe, Nicola Atkinson, Wendy A. Harwood

**Affiliations:** Department of Crop Genetics, John Innes Centre, Norwich Research Park, Norwich, Norfolk NR4 7UH UK

## Correction to: Plant Methods (2019) 15:121 10.1186/s13007-019-0503-z

In the original publication of the article [1], a few errors were identified.

In the Materials and methods section, under the subheading “Immature embryos isolation”, the term 0.44 mg L^−1^ should read as 0.44 g L^−1^.

Under the subheading, “Resting period, callus induction and selection of transformed material” the term 5 mg L^−1^ and 15 mg mL^−1^ should read as 5 g L^−1^ and 15 mg L^−1^.

Under the subheading “Regeneration of transgenic plants” the term 20 mg L^−1^ and 0.5 mg L^−1^ should read as 20 g L^−1^ and 0.5 g L^−1^ .

In the Results section, under the subheading “Optimization of wheat transformation with Agrobacterium Effect of gelling agent on callus induction and regeneration”, the term 2%, 5%, 3.5% and 8% should read as 0.2%, 0.5%, 0.35% and 0.8%.
